# Kinetic classification of posterior semicircular canal canalithiasis based on quantitative nystagmus parameters and its predictive value for the need of a second Epley maneuver: a retrospective quantitative analysis of 60 patients

**DOI:** 10.3389/fneur.2026.1881896

**Published:** 2026-07-28

**Authors:** Jing Chen, Shuo Yang

**Affiliations:** Zhengzhou Central Hospital Affiliated to Zhengzhou University, Zhengzhou, China

**Keywords:** benign paroxysmal positional vertigo, Epley maneuver, inhibitory nystagmus, second repositioning, slow phase velocity

## Abstract

**Objective:**

To classify the kinetic state of otoconia in canalithiasis according to the slow phase velocity (SPV) of nystagmus during the Dix‑Hallpike test in patients with posterior semicircular canal benign paroxysmal positional vertigo (PSC‑BPPV), and to evaluate the association between different subtypes and the need for a second Epley maneuver, thereby establishing a prognostic model based on kinetic classification.

**Methods:**

Clinical data of 60 patients with unilateral PSC canalithiasis were retrospectively analyzed. Receiver operating characteristic (ROC) curves were used to determine the optimal threshold of diagnostic vertical SPV for predicting the need for a second repositioning. Patients were subsequently divided into a high‑SPV aggregated type and a low‑SPV dispersed type based on better prognostic discrimination in clinical grouping (threshold = 20%). The direction and SPV of nystagmus during each step of the Epley maneuver were recorded. The primary outcome was a positive Dix‑Hallpike test 24–48 hours after the initial maneuver, indicating the need for a second repositioning. The rates of second repositioning and the characteristics of inhibitory nystagmus during the maneuver were compared between the two types. Logistic regression was used to analyze the independent predictive value of the classification.

**Results and discussion:**

ROC curve analysis showed that the area under the curve (AUC) of diagnostic vertical SPV for predicting a second repositioning was 0.85 (95% CI 0.76–0.94, *p* < 0.001). 21 patients (35.0%) were classified as high‑SPV aggregated type (vertical SPV ≥20°/s) and 39 patients (65.0%) as low‑SPV dispersed type (vertical SPV <20°/s). The rate of second repositioning was 14.3% (3/21) in the high‑SPV aggregated type and 41.0% (16/39) in the low‑SPV dispersed type (*χ*^2^ =, *p* =0.041). Among patients with inhibitory nystagmus at step 4, the low‑SPV dispersed type had a high second‑session rate of 84.6% (11/13). Multivariate logistic regression showed that both the low‑SPV dispersed type (OR=4.7, 95% CI 1.2–19.3, *p* = 0.031) and inhibitory nystagmus at step 4 (OR=14.5, 95% CI 3.1–68.2, *p* < 0.001) were independent predictors of the need for a second repositioning. The events-per-variable ratio was approximately 9.5:1, which is acceptable but relatively modest; thus, the results warrant cautious interpretation. These findings are exploratory and require validation in independent external cohorts.

## 1 Introduction

Benign paroxysmal positional vertigo (BPPV) is the most common peripheral vestibular disorder, and posterior semicircular canal BPPV accounts for 70–90% of all cases (1, 2). The Epley canalith repositioning maneuver is the first-line treatment for PSC-BPPV, with a single- or multiple-session success rate of about 70–90% (3); nevertheless, a considerable proportion of patients require a second or even more repositioning sessions. How to predict the long-term outcome during the maneuver itself and identify difficult cases that may need a second repositioning is an urgent clinical issue.

Classic theory states that canalithiasis results from detached otoconial particles that enter the semicircular canal lumen and move freely. When the head moves in the plane of the affected semicircular canal, the otoconia move under gravity, generate endolymph flow, stimulate the cupula, and produce positional vertigo and nystagmus (4). However, there are significant individual differences in the size, number, and physical state of the detached otoconia, which may directly affect the movement behavior of the otoconia and the difficulty of repositioning.

Domínguez-Durán et al. (5) conducted a prospective multicenter study of 115 patients with PSC-BPPV and found that nystagmus intensity was one of the most valuable clinical variables for predicting successful Epley repositioning, ranking together with a history of ipsilateral BPPV as the two most important prognostic factors. Lou et al. (6) studied 276 patients and further pointed out that excitatory nystagmus (i.e., consistent with the diagnostic phase) at the third step of the Epley maneuver had a sensitivity of 88.9% for treatment success, whereas inhibitory nystagmus (i.e., reversing with the diagnostic phase) or absence of nystagmus indicated a poor outcome. We define nystagmus that does not change direction during the Epley maneuver as “excitatory nystagmus” (consistent with ampullopetal endolymph flow), and nystagmus that reverses direction during the Epley maneuver as “inhibitory nystagmus” (consistent with ampullofugal endolymph flow). Moreover, some studies have suggested that residual dizziness after successful repositioning may be related to the presence of tiny otoconia that are insufficient to provoke obvious nystagmus (7).

Although the above studies emphasize the prognostic value of the qualitative direction and quantitative intensity of nystagmus, no study has systematically integrated diagnostic quantitative SPV parameters with inhibitory nystagmus during the repositioning maneuver to construct a kinetic classification of canalithiasis. Based on this, the present study proposes the following classification hypothesis:

*High SPV aggregated type*: characterized by higher vertical SPV, presumably reflecting large, well-aggregated otoconia that are more readily cleared in a single maneuver;

*Low-SPV dispersed type*: characterized by lower vertical SPV, presumably reflecting small, dispersed particles that are more likely to leave residual debris and require multiple sessions.

We used ROC curves to determine the optimal threshold, verified the predictive performance of this classification for the need of a second repositioning, and analyzed differences in inhibitory nystagmus characteristics during the Epley maneuver between the two types.

## 2 Materials and methods

### 2.1 Study population

Consecutive patients with unilateral PSC-BPPV who attended the Vertigo Center of our hospital from August 2024 to December 2025 were enrolled. Inclusion criteria: (1) typical up-beating torsional nystagmus elicited by the Dix-Hallpike test, consistent with a diagnosis of PSC canalithiasis (8); (2) complete recording of diagnostic nystagmus parameters and the direction and SPV of nystagmus during all steps (2, 3, and 4) of the Epley maneuver using video-nystagmography (VNG); (3) return for a follow-up Dix-Hallpike test 24–48 h after the initial maneuver, with clear documentation of whether a second repositioning was performed. Exclusion criteria: (1) multi-canal BPPV or coexisting central vestibular disorders; (2) previous repositioning treatment for the current episode; (3) incomplete clinical data. A total of 60 patients were finally analyzed, including 28 males and 32 females, with ages ranging from 32 to 85 years (median 57.5 years). This study was approved by the Institutional Review Board of the Affiliated Zhengzhou Central Hospital of Zhengzhou University.

### 2.2 Nystagmus recording and repositioning procedure

All patients were examined with the same VNG device (Chartr VNG). Diagnostic phase: the patient sat with the head turned 45 ° toward the affected side, then was rapidly lowered to the supine position with the head hanging 30 °; latency, duration, maximum vertical SPV, and maximum horizontal SPV were recorded. Subsequently, a standardized Epley maneuver was performed by the same senior technician, each position being maintained until nystagmus ceased or for at least 30 s. During the entire repositioning, VNG recorded in real time the direction and corresponding peak SPV of nystagmus at step 2 (head rotated 90 ° to the healthy side), step 3 (body rolled to the healthy side), and step 4 (patient slowly brought to a sitting position). No immediate retesting was performed; all patients returned for a follow-up Dix-Hallpike test 24–48 h later to decide whether a second repositioning was needed.

### 2.3 Definition of kinetic classification

In this study, we classified nystagmus based on whether its direction was consistent with the excitatory response of the affected semicircular canal during the diagnostic Dix-Hallpike positioning:

*Excitatory nystagmus*: nystagmus direction is consistent with excitation of the affected semicircular canal (i.e., ampullopetal endolymph flow). In practical terms, this means the nystagmus direction does not change during the Epley maneuver relative to the diagnostic phase (e.g., up-beating and torsional toward the affected ear).

*Inhibitory nystagmus*: nystagmus direction is consistent with inhibition of the affected semicircular canal (i.e., ampullofugal endolymph flow). In practical terms, this means the nystagmus direction reverses during the Epley maneuver relative to the diagnostic phase (e.g., down-beating and torsional toward the healthy ear).

*Down-beating nystagmus*: nystagmus with a dominant vertical downward component; the torsional component may be either consistent with the excitatory response or indistinct.

*Horizontal nystagmus*: nystagmus predominantly horizontal in direction.

*Persistent inhibitory nystagmus*: inhibitory nystagmus lasting more than 10 s or appearing continuously across multiple steps.

Receiver operating characteristic (ROC) curves were used to determine the optimal threshold of diagnostic vertical SPV for predicting a second repositioning (see Section 2.4 and results 3.2). Based on this threshold, patients were divided into two types:

*High-SPV aggregated type*: vertical SPV ≥ threshold (20 °/s), Large aggregated otoconia, strong gravitational driving force, High single-session success rate.

*Low-SPV dispersed type*: vertical SPV < threshold (20 °/s), Small dispersed otoconial particles, weak gravitational driving force, Prone to residual debris, high risk of multiple sessions.

### 2.4 Statistical analysis

SPSS 26.0 software was used. Continuous data were expressed as median (interquartile range) and compared between groups using the Mann–Whitney *U* test; categorical data were expressed as counts (percentages) and compared using the *χ*^2^ test or Fisher’s exact test.

*ROC curve analysis steps*: with “need for second repositioning” (yes = 1, no = 0) as the state variable and diagnostic vertical SPV as the test variable, ROC Curve was performed. “ROC curve,” “diagonal reference line,” and “coordinates of the curve” were selected. From the output coordinate table, the Youden index (Youden index = sensitivity + specificity − 1) was calculated for each cutoff value, and the cutoff with the maximum Youden index was selected as the optimal threshold. The area under the curve (AUC) and its 95% confidence interval were also reported.

Univariate and multivariate logistic regression (forward stepwise, likelihood ratio) were used to analyze the relationship between the kinetic classification, inhibitory nystagmus at step 4, and other potential confounding factors with the need for a second repositioning. Odds ratios (OR) and 95% confidence intervals (CI) were calculated. A *p*-value <0.05 was considered statistically significant.

Given the limited sample size (60 patients, 19 outcome events), the multivariate logistic regression model included 2 pre-specified key variables (kinetic type and step 4 inhibitory nystagmus) to avoid overfitting. The events-per-variable ratio was approximately 9.5:1, which meets the rule of thumb (generally requiring ≥5–10) but is relatively modest. Univariate and multivariate logistic regression (forward stepwise, likelihood ratio) were used to analyze the relationship between the kinetic classification, inhibitory nystagmus at step 4, and other potential confounding factors with the need for a second repositioning. Odds ratios (OR) and 95% confidence intervals (CI) were calculated. A *p*-value <0.05 was considered statistically significant.

## 3 Results

### 3.1 Baseline characteristics and distribution of kinetic types

Among the 60 patients, 21 (35.0%) were classified as high-SPV aggregated type and 39 (65.0%) as low-SPV dispersed type. There were no statistically significant differences in age, sex, or affected side between the two types (*p* > 0.05), indicating comparability. Diagnostic nystagmus parameters of the two types are compared in Table 1.

**Table 1:** Comparison of diagnostic nystagmus parameters between the two kinetic types.

Parameter	High-SPV aggregated type (*n* = 21)	Low-SPV dispersed type (*n* = 39)	*p*-value
Max vertical SPV [°/s, *M* (IQR)]	32.4 (26.5–45.4)	9.2 (5.4–14.9)	<0.001
Max horizontal SPV [°/s, *M* (IQR)]	8.3 (6.1–12.8)	6.5 (4.2–8.4)	0.091
Latency [s, *M* (IQR)]	2 (1–3)	4 (2–7)	0.002
Duration [s, *M* (IQR)]	15 (10–24)	12 (7–21)	0.087

### 3.2 ROC curve analysis for optimal vertical SPV threshold

ROC analysis with “need for second repositioning” as the state variable and diagnostic vertical SPV as the test variable gave an AUC of 0.85 (95% CI 0.76–0.94, *p* < 0.001). Sensitivity, specificity, and Youden indices for different cutoffs are shown in Table 2. The Youden index was highest (59.4%) at a cutoff of 15°/s. However, the Youden index at 20 °/s was 59.1%, only 0.3 percentage points lower. We selected 20 °/s as the primary threshold because: (1) it provided better prognostic discrimination in clinical grouping (*p* = 0.041 for comparison of second-session rates between the two types), whereas the 15°/s cutoff did not (*p* = 0.232). At this cutoff, sensitivity was 73.7% (14/19) and specificity 85.4% (35/41).

**Table 2:** Predictive performance of different diagnostic vertical SPV cutoffs.

Cutoff (°/s)	Sensitivity (%)	Specificity (%)	Youden index (%)
10	89.5	63.4	52.9
15	78.9	80.5	59.4
20	73.7	85.4	59.1
25	63.2	90.2	53.4
30	47.4	95.1	42.5

### 3.3 Comparison of second-session rates between the two types

The rate of second repositioning was 14.3% (3/21) in the high-SPV aggregated type and 41.0% (16/39) in the low-SPV dispersed type, and the difference was statistically significant (*χ*^2^ = 4.13, *p* = 0.041). Patients with the low-SPV dispersed type had a significantly higher risk of needing a second repositioning (Table 3).

**Table 3:** Comparison of second-session rates between the two kinetic types.

Kinetic type	Second session needed [*n* (%)]	Second session not needed [*n* (%)]	Total
High-SPV aggregated	3 (14.3)	18 (85.7)	21
Low-SPV dispersed	16 (41.0)	23 (59.0)	39
Total	19 (31.7)	41 (68.3)	60

### 3.4 Comparison of nystagmus features between the two types

The SPV of inhibitory nystagmus at step 4 was significantly higher in the low-SPV dispersed type than in the high-SPV aggregated type (*p* = 0.023), whereas the incidence of inhibitory nystagmus at each step did not differ significantly between the groups (Table 4).

**Table 4:** Comparison of inhibitory nystagmus characteristics during repositioning between the two types.

Parameter	High-SPV aggregated (*n* = 21)	Low-SPV dispersed (*n* = 39)	*p*-value
Inhibitory nystagmus at step 3 [*n* (%)]	4 (19.0)	10 (25.6)	0.560
Inhibitory nystagmus at step 4 [*n* (%)]	4 (19.0)	13 (33.3)	0.236
Inhibitory nystagmus at step 3 or 4 [*n* (%)]	5 (23.8)	17 (43.6)	0.126
SPV of step 4 inhibitory nystagmus [°/s, *M* (IQR)]	8.5 (6–11)	12 (9–18)	0.023

### 3.5 Combined predictive performance of kinetic type and step 4 inhibitory nystagmus

Among patients with the low-SPV dispersed type, those with step 4 inhibitory nystagmus (*n* = 13) had a second-session rate of 84.6% (11/13), whereas those without step 4 inhibitory nystagmus (*n* = 26) had a rate of 19.2% (5/26) (*χ*^2^ = 15.38, *p* < 0.001). Among patients with the high-SPV aggregated type, the second-session rate was 25.0% (1/4) in those with step 4 inhibitory nystagmus and 11.8% (2/17) in those without; the difference was not statistically significant (*p* = 0.483, Fisher’s exact test) (see Table 5).

**Table 5:** Rate of second repositioning according to step 4 inhibitory nystagmus within each kinetic type.

Kinetic type	Step 4 inhibitory nystagmus (present)	Step 4 inhibitory nystagmus (absent)
High-SPV aggregated	1/4 (25.0%)	2/17 (11.8%)
Low-SPV dispersed	11/13 (84.6%)	5/26 (19.2%)
Total	12/17 (70.6%)	7/43 (16.3%)

### 3.6 Multivariate logistic regression analysis

Using “need for a second repositioning” as the dependent variable, kinetic type, step 4 inhibitory nystagmus, latency (≤2 s considered abnormal), and duration (continuous) were entered into a multivariate logistic regression model (forward stepwise). The results showed that both *low-SPV dispersed type* (OR = 4.7, 95% CI 1.2–19.3, *p* = 0.031) and step 4 inhibitory nystagmus (OR = 14.5, 95% CI 3.1–68.2, *p* < 0.001) were independent predictors of the need for a second repositioning (Table 6). The events-per-variable ratio was approximately 9.5:1, which is acceptable but relatively modest; thus, the results should be interpreted with caution.

**Table 6:** Multivariate logistic regression analysis for predicting the need for a second repositioning.

Variable	*β*	Wald *χ*^2^	OR	95% CI	*p*-value
Low-SPV dispersed (vs. high-SPV aggregated)	1.54	4.66	4.7	1.2–19.3	0.031
Step 4 inhibitory nystagmus	2.68	13.21	14.5	3.1–68.2	<0.001
Constant	−3.10	18.95	0.045	—	<0.001

## 4 Discussion

### 4.1 Pathophysiological basis of the vertical SPV kinetic classification and threshold selection

Using ROC analysis, we identified the optimal cutoff of diagnostic vertical SPV for predicting a second repositioning as 20°/s (AUC = 0.85). This cutoff allowed us to classify patients into high-SPV aggregated and low-SPV dispersed types, a classification that aligns with the basic biomechanical principles of canalithiasis. High SPV usually corresponds to large, well-aggregated otoconial masses, which move rapidly under gravity, generate strong endolymph flow, and are therefore more likely to be cleared in a single Epley maneuver. In contrast, low SPV generally reflects small, dispersed otoconial particles that move slowly and tend to float, resulting in a high risk of residual debris after repositioning.

This result is highly consistent with the conclusion of Domínguez-Durán et al. (5), who emphasized that nystagmus intensity is one of the most important clinical predictors of Epley maneuver success. Our study further quantifies this qualitative conclusion by providing a specific SPV cutoff, thereby enhancing clinical operability.

### 4.2 Different prognostic implications of inhibitory nystagmus in the two kinetic types

We found that the SPV of step 4 inhibitory nystagmus was significantly higher in the low-SPV dispersed type than in the high-SPV aggregated type (12 °/s vs. 8.5 °/s, *p* = 0.023), and when low-SPV dispersed type was combined with step 4 inhibitory nystagmus, the second-session rate reached 84.6%. In contrast, step 4 inhibitory nystagmus was not significantly associated with outcome in the high-SPV aggregated type. This difference suggests that step 4 inhibitory nystagmus in low-SPV dispersed patients is more likely a genuine sign of treatment failure. The mechanism might be that small, dispersed otoconia produce unfavorable retrograde endolymph flow (ampullopetal flow) during the final part of the maneuver, leading to incomplete clearance. In high-SPV aggregated patients, inhibitory nystagmus at step 4 may only represent a brief physiological disturbance as the otoconia enter the utricle, rather than heralding treatment failure.

Lou et al. (6) reported that the appearance of inhibitory nystagmus or absence of nystagmus during the Epley maneuver was associated with a significantly lower success rate. Our study further reveals that the prognostic significance of inhibitory nystagmus is modulated by the diagnostic SPV level; it acquires strong failure-predicting value only when the patient also has low SPV.

### 4.3 Proposed clinical decision pathway

Based on the above findings, we propose the following individualized management strategy:

*Low-SPV dispersed type with obvious step 4 inhibitory nystagmus (SPV ≥ 20 °/s)*: patients should be clearly informed of a high probability (>80%) of needing a second repositioning. They can be scheduled for an early follow-up within 24–48 h, or immediate repeat repositioning (consider switching to the Semont maneuver if necessary) can be considered.

*High-SPV aggregated type*: even if step 4 inhibitory nystagmus occurs, routine follow-up (5–7 days) can be adopted to avoid unnecessary early intervention.

### 4.4 Comparison and integration with previous studies

Previous literature has focused either on nystagmus direction alone (6) or on nystagmus intensity alone (5), the present study is the first to combine diagnostic quantitative SPV with nystagmus direction and intensity during the repositioning maneuver, constructing a binary kinetic classification. This classification not only explains why some patients with nystagmus have good outcomes while others have poor outcomes, but also provides a quantifiable stratification tool for clinical use. Furthermore, our results echo the hypothesis of Seok et al. (7) about residual dizziness and residual microscopic debris, suggesting that the low-SPV dispersed type may be precisely the population at high risk of residual debris.

### 4.5 Limitations

This is a single-center retrospective study with a limited sample size (60 patients), especially only 21 patients in the high-SPV aggregated type, which restricts in-depth subgroup analysis. The ROC-determined cutoff of 20 °/s is based on this dataset and has not been validated in an independent external cohort, so overfitting is possible. The kinetic classification relies solely on vertical SPV, a single parameter; future studies could incorporate multiple parameters such as nystagmus decay time and the horizontal-to-vertical SPV ratio for combined classification. In addition, all nystagmus data were collected with the same VNG device; multi-device consistency has not been tested. These limitations need to be addressed in future prospective, multicenter studies.

## 5 Conclusion

The kinetic classification, comprising the high-SPV aggregated type and the low-SPV dispersed type based on a diagnostic vertical SPV cutoff of 20 °/s, may be useful for predicting the need for a second Epley maneuver in patients with PSC canalithiasis. Patients with the low-SPV dispersed type have a significantly higher risk of requiring a second repositioning and are more likely to exhibit high-intensity inhibitory nystagmus at step 4. This classification provides an objective, quantitative basis for individualized treatment decisions.

## Data Availability

The original contributions presented in the study are included in the article/supplementary material, further inquiries can be directed to the corresponding author.

## References

[1] von Brevern M Radtke A Lezius F Feldmann M Ziese T Lempert T . Epidemiology of benign paroxysmal positional vertigo: a population based study. J Neurol Neurosurg Psychiatry. (2007) 78:710–5. doi: 10.1136/jnnp.2006.100420, 17135456 PMC2117684

[2] Bhattacharyya N Gubbels SP Schwartz SR Edlow JA El-Kashlan H Fife T . Clinical practice guideline: benign paroxysmal positional vertigo (update). Otolaryngol Head Neck Surg. (2017) 156:S1–S47. doi: 10.1177/0194599816689667, 28248609

[3] Woodworth BA Gillespie MB Lambert PR. The canalith repositioning procedure for benign positional vertigo: a meta-analysis. Laryngoscope. (2004) 114:1143–6. doi: 10.1097/00005537-200407000-00002, 15235337

[4] Hall SF Ruby RR McClure JA. The mechanics of benign paroxysmal vertigo. J Otolaryngol. (1979) 8:151–8. 430582

[5] Domínguez-Durán E Domènech-Vadillo E Álvarez-Morujo de Sande MG González-Aguado R Guerra-Jiménez G Ramos-Macías Á . Analysis of risk factors influencing the outcome of the Epley maneuver. Eur Arch Otorrinolaringol. (2017) 274:3567–76. doi: 10.1007/s00405-017-4674-9, 28725982

[6] Lou Y Xu L Wang Y Zhao Z Liu X Li Y. Orthotropic nystagmus in predicting the efficacy of treatment in posterior canal benign paroxysmal positional vertigo. Am J Otolaryngol. (2020) 41:102472. doi: 10.1016/j.amjoto.2020.102472, 32276733

[7] Seok JI Lee HM Yoo JH Lee DK. Residual dizziness after successful repositioning treatment in patients with benign paroxysmal positional vertigo. J Clin Neurol. (2008) 4:107–10. doi: 10.3988/jcn.2008.4.3.107, 19513312 PMC2686873

[8] Dix MR Hallpike CS. The pathology symptomatology and diagnosis of certain common disorders of the vestibular system. Proc R Soc Med. (1952) 45:341–54. doi: 10.1177/003591575204500604, 14941845 PMC1987487

